# Emerging approaches for detection of methylation sites in RNA

**DOI:** 10.1098/rsob.180121

**Published:** 2018-09-05

**Authors:** Anna Ovcharenko, Andrea Rentmeister

**Affiliations:** Institute of Biochemistry, Department of Chemistry, University of Münster, Wilhelm-Klemm-Straße 2, D-48149 Münster, Germany

**Keywords:** RNA methylation, m^6^A, site-specific methylation detection

## Abstract

RNA methylations play a significant regulatory role in diverse biological processes. Although the transcriptome-wide discovery of unknown RNA methylation sites is essential to elucidate their function, the development of a bigger variety of detection approaches is desirable for multiple reasons. Many established detection methods for RNA modifications heavily rely on the specificity of the respective antibodies. Thus, the development of antibody-independent transcriptome-wide methods is beneficial. Even the antibody-independent high-throughput sequencing-based methods are liable to produce false-positive or false-negative results. The development of an independent method for each modification could help validate the detected modification sites. Apart from the transcriptome-wide methods for methylation detection *de novo*, methods for monitoring the presence of a single methylation at a determined site are also needed. In contrast to the transcriptome-wide detection methods, the techniques used for monitoring purposes need to be cheap, fast and easy to perform. This review considers modern approaches for site-specific detection of methylated nucleotides in RNA. We also discuss the potential of third-generation sequencing methods for direct detection of RNA methylations.

## 1. Introduction

Nowadays, the importance of RNA modifications and RNA methylation in particular is evident. To date, 171 RNA modifications are known according to the MODOMICS database, of which 72 include methyl groups [1]. The internal modifications are present in different RNA classes, such as tRNA, rRNA, mRNA, snRNA, lncRNA as well as in viral RNA genomes. The biological functions of RNA methylation greatly vary depending on the modified nucleoside and the RNA type. Thus, some bacterial rRNA methylations are responsible for the macrolide antibiotic resistance [2]. Methylation in eukaryotic mRNA is considered to play a significant role in posttranscriptional regulation [3]. However, not all the biological roles have been clarified to date.

RNA was shown to contain methylated bases in 1958 [4], and many important discoveries were made in the following years regarding the localization and biosynthesis of methylation in RNA. However, the lack of sensitive methods for detection of RNA modifications together with the common conception of their static and non-reversible nature led to restrained scientific interest in this field. For many years, the RNA modifications stayed in the shadow of extensively studied DNA and protein modifications. The discovery that the methyl group of *N*^6^-methyladenosine (m^6^A) is also removable and hence an RNA modification can be reversible sparked broad scientific attention in the past few years. An exciting hypothesis about the reversibility of m^6^A was proposed, stating that its levels would be mediated not only by its writers, but also by its erasers, removing this modification in a dynamic manner [5]. Although this concept has also been criticized [6], the kindled interest in RNA modifications triggered the development of many approaches for their precise mapping. Apart from m^6^A, also 1-methyladenosine (m^1^A) was recently discovered to be reversible in tRNA and mRNA [7]. Accessibility of next-generation sequencing (NGS) technologies led to the development of the precise transcriptome-wide mapping methods of modified RNA nucleotides.

**Figure 1.**
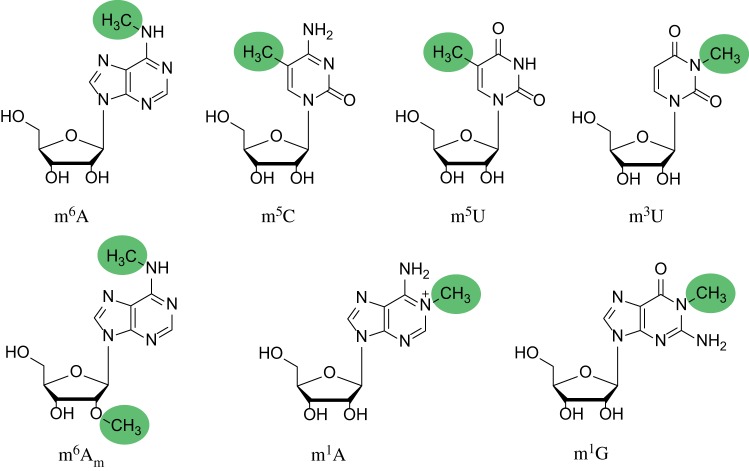
RNA methylations mentioned in the current review. *N*^6^-methyladenosine (m^6^A), 5-methylcytidine (m^5^C), 5-methyluridine (m^5^U), 3-methyluridine (m^3^U), *N*^6^,2′-*O*-dimethyladenosine (m^6^A_m_), 1-methyladenosine (m^1^A) and 1-methylguanosine (m^1^G).

In the current review, we focus on recent advances in the field of methylation detection in RNA and discuss innovative approaches for site-specific detection of methylation, including the ones that still need further development. We will also discuss approaches for monitoring the presence of methylated nucleotides at a specific position in RNA. We will not address the well-established methods for transcriptome-wide mapping of RNA modifications coupled to NGS, because they have been extensively reviewed elsewhere [8,9]. Figure 1 shows the chemical structures of RNA methylations most frequently mentioned in the current review.

## 2. Historical overview

Early methods for detection and quantification of modified nucleosides/nucleotides were based on their physico-chemical properties. The separation of RNA monomers was performed using TLC (thoroughly reviewed by Grosjean *et al*. [10]) or by high-performance liquid chromatography (HPLC) coupled with UV detection or/and mass spectrometry (MS). To improve the quantification of the modified nucleosides with LC–MS, stable isotope labelling approaches were developed [11–13]. The classic LC–MS analysis of RNA modifications involves RNA digestion to single nucleosides, which causes loss of the sequence context. This can be overcome by separate enzymatic digestion of analysed RNA sample to nucleosides and oligonucleotides and analysis of both datasets. This approach was used to site-specifically locate modifications in the tRNA sequence [14]. Direct label-free localization of m^6^A, 5-methylcytidine (m^5^C), 3-methyluridine (m^3^U) and 5-methyluridine (m^5^U) in short synthetic oligoribonucleotides was recently shown to be possible with a mass spectrometer equipped for low-energy collisionally activated dissociation experiments [15].

The early approaches for precise localization of RNA modifications relied on the RNase digestion and fingerprinting in combination with paper electrophoresis and TLC. For example, the m^6^A sites in Rous sarcoma virus RNA were already mapped in 1985 [16].

Another group of approaches for methylation detection in RNA is based on reverse transcription (RT). The detection of modified RNA nucleotides is possible if their presence influences the reverse transcriptase either by blocking or stalling RT, or by inducing misincorporation into cDNA opposite the modification [17]. The importance of the RT-based methods has significantly increased, because they can be directly linked to NGS allowing for transcriptome-wide analysis. The well-established methods for RNA modification analysis that use NGS technologies have been described in an excellent recent review by Schwartz & Motorin [8].

A remarkable method for m^6^A detection at single-nucleotide resolution is based on the combination of RNase H site-specific cleavage, splinted ligation, ribonuclease digestion and TLC (site-specific cleavage and radioactive-labelling followed by ligation-assisted extraction and TLC, or SCARLET) [18]. In principle, SCARLET can also be used to detect other RNA modifications than m^6^A. However, this method is extremely laborious and time-consuming.

In the current review, we focus on recent advances in the field of site-specific detection of methylation in RNA. The approaches are broadly categorized based on their working principle.

## 3. Immunochemical approaches

Antibodies are commercially available for many methylated RNA residues, such as m^6^A, m^1^A and m^5^C. Modification-specific antibodies in conjunction with NGS and (optionally) cross-linking were used to map m^5^C, m^1^A and m^6^A at the transcriptome-wide level [19–22]. However, the reliability of those antibodies is still questionable. The m^6^A-specific antibody was shown not to distinguish between m^6^A and *N*^6^,2′-*O*-dimethyladenosine (m^6^A_m_) [23]. Moreover, the modification-specific antibodies might introduce bias due to off-target binding, and highly structured nucleic acids might impede the antibody–antigen interactions [24].

The application of methylation-specific antibodies is not limited to transcriptome-wide detection in combination with NGS. They have also been used for immuno-northern blotting [25]. Recently, also several electrochemical and chemical biosensors for m^6^A detection were developed, which, however, do not provide information about the m^6^A position in the sequence [26].

Another recently reported immunochemical approach is based on the immunorecognition of m^6^A in a bulge loop of an RNA–DNA duplex. The m^6^A is specifically recognized by an antibody in the one-bulge loop, but not in a fully matched RNA–DNA hybrid. The biotinylated DNA probe was designed to target an m^6^A-containing region, but lacked a nucleotide pairing with m^6^A*.* The developed approach was applied to detect m^6^A_2030_ in 23S rRNA of *Escherichia coli* total RNA*.* A significant difference between the one-bulge-inducing probe and the full-match probe is achieved in case of prior RNA fragmentation. However, the approach does not allow to achieve specificity in intact RNA samples. In addition, high background signals are observed in a control experiment for a non-methylated adenosine [27].

## 4. Approaches based on methylation-sensitive enzymes

Some of the methylated RNA nucleotides naturally block RT, facilitating the development of RT-based methods. In case the methyl group is present on the Watson–Crick edge (m^1^A, m^3^U, 3-methylcytidine (m^3^C) and 1-methylguanosine (m^1^G)), the base-pairing and therefore the RT signatures can be affected. One example of the established specific RT signatures for methylated RNA residues is m^1^A [28].

Since different RT enzymes can have different sensitivity for methylation of RNA, it is possible to affect the RT signature by varying the enzyme and the reaction conditions used for RT, such as the buffer composition, or the dNTP concentration. Thus, a specialized protocol for detecting 2′-O-methylation in RNA was developed for a low dNTP concentration [29]. For m^6^A, another RNA modification previously considered RT silent, a selective polymerase enabling its detection was identified. A polymerase from *Thermus thermophilus* with RT activity was selective by up to 18-fold for incorporation of thymidine opposite adenosine over m^6^A [30].

**Figure 2.**
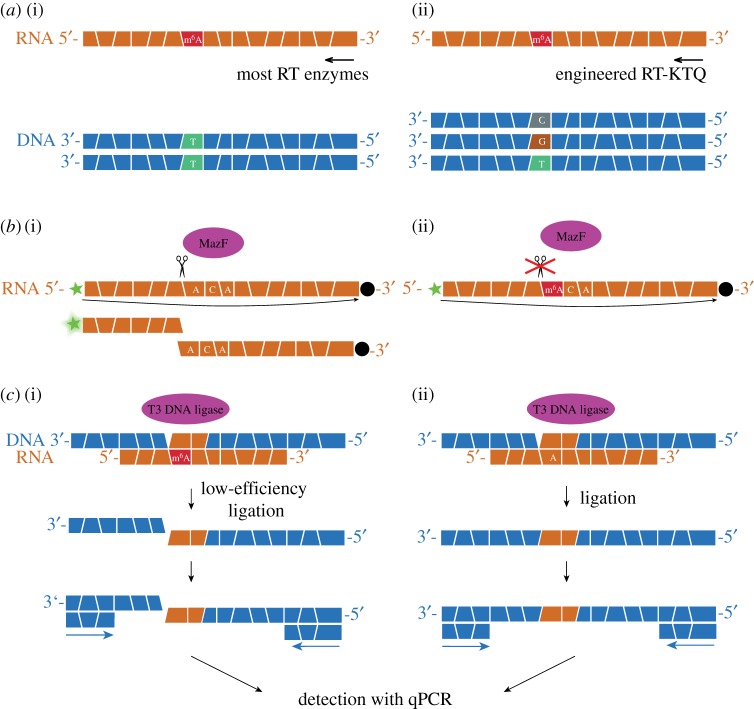
Methylation-sensitive enzymes to detect RNA methylation. (*a*) (i) The modification m^6^A is silent for most RT enzymes. (ii) Protein engineering led to the development of an m^6^A-sensitive enzyme variant, which introduces misincorporations opposite m^6^A [32]. (*b*) MazF endoribonuclease cleaves unmethylated 5′-ACA-3′ sequences (i), but not 5′-(m^6^A)CA-3′ sequences (ii). The oligonucleotide has a 6-FAM fluorophore at the 5′-end (shown as a green star) and a BHQ1 quencher at the 3′-end (shown as black disc). MazF cleavage leads to the increase of fluorescence signal due to the separation of fluorophore and quencher [33]. (*c*) m^6^A sensitivity of the T3 DNA ligase is used for m^6^A detection via qPCR. For this, two DNA probes are hybridized adjacent to the analysed adenosine. One of the probes is modified at the 3′-end with two ribonucleotides. Owing to the different ligation efficiency for A- and m^6^A-containing RNA, the presence of m^6^A can be detected with the qPCR of the ligation product [34].

Detection of RT-silent modified RNA residues can be improved by engineering the reverse transcriptase enzymes to introduce signatures opposite the modification. Aschenbrenner *et al.* evolved both 2′-*O*-methyl- and m^6^A-sensitive polymerases from a thermostable KlenTaq variant [31,32]. In case of 2′-*O*-methylation, stalling of the RT by the engineered enzyme was employed in a methylation-sensitive qRT-PCR and was used for quantification of 2′-*O*-methylation in human 18S rRNA from different cell types. For m^6^A, the aim of DNA polymerase engineering was not only to induce blocking of the RT, but also to introduce signatures opposite the m^6^A (figure 2*a*). The evolved enzyme variant was applied to the NGS protocol for the analysis of a known m^6^A site in *E. coli* tRNA^Val^. An error rate of 14.3% was observed at the known m^6^A site. However, the misincorporation with error rates greater than 10% was also observed opposite 5-methyluridine and at the 5′-end of the RNA molecule. This suggests that the engineered enzyme is sensitive also to RNA modifications other than m^6^A, and the developed approach is ineffective for m^6^A detection at the 5′-end of the molecule, where the rates are inaccurate due to the low coverage.

Proteins other than DNA polymerases can also be sensitive to RNA methylation, including RNA-binding proteins (such as human single-stranded RNA-binding protein Pumilio 2 [35]), endoribonucleases and DNA ligases. *Escherichia coli* MazF toxin was recently identified as the first m^6^A-sensitive RNA cleavage enzyme [33]. This endoribonuclease was shown to cleave RNAs within an 5′-ACA-3′ sequence motif, but not within 5′-(m^6^A)CA-3′. Based on this finding, a FRET-based assay was developed to determine the methylation status of RNA (figure 2*b*). However, it should be noted that MazF was also sensitive to m^1^A and is thus not suitable for distinguishing between methylation at those two positions. In addition, MazF does not cleave double-stranded sequences, thus hindering m^6^A detection in a structured RNA [33].

The first developed ligation-based method for m^6^A detection allowed discrimination between A and m^6^A, when T4 DNA ligase was used [36]. Recently, the T3 DNA ligase was identified to have much stronger selectivity to discriminate A from m^6^A. On the basis of this discovery, a PCR-based approach was developed for m^6^A detection [34]. Two DNA probes adjacently hybridize with RNA around the target adenosine residue. In the case of *N*^6^-methylation, the ligation is significantly hindered, which can be detected in the quantitative PCR (qPCR) of the ligation product. For better discrimination between A and m^6^A, one of the probes should be modified with two ribonucleotides, as shown in figure 2*c*. The method also enables the quantification of the fraction of RNA containing m^6^A at this position. This quantification was validated for one of the known m^6^A sites in MALAT1 lncRNA in the polyA^+^ RNA isolated from different cell types.

## 5. Approaches based on hybridization properties

**Figure 3.**
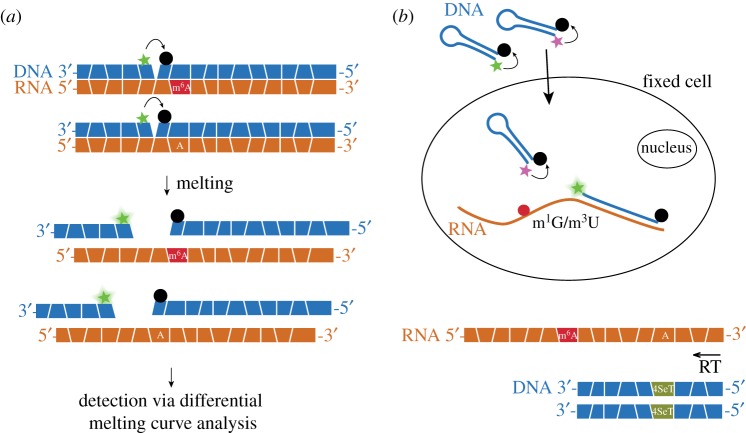
Hybridization properties as basis to detect RNA methylation. (*a*) The presence of m^6^A changes the melting properties of an RNA–DNA duplex. The m^6^A detection requires two DNA probes complementary to the analysed region, carrying a fluorophore (shown as green star) and a quencher (black disc). Differential analysis of the melting curves allows for m^6^A detection [39]. (*b*) Molecular beacons can be used as methylation-sensitive hybridization probes in fixed cells. Methylation types such as m^1^G and m^3^U destabilize the base-pairing, which prevents the probe from binding to the RNA. A methylation-insensitive probe carrying a different fluorophore and complementary to another region of the analysed RNA molecule serves as an internal control [40]. (*c*) m^6^A sites can be detected via the stalled RT of an m^6^A-containing RNA in case selenium-containing dTTP analogues are used [41].

Even if methylation in RNA is present on the Watson–Crick edge, it does not necessarily impede base-pairing. A prime example is m^6^A, which still forms the A–U base-pair including the hydrogen bond between the *N*^6^-position of adenosine and O4 in uridine. Hence, the presence of m^6^A reduces the thermodynamic stability of RNA duplexes likely due to the steric hindrance [37], but stabilizes single-stranded RNA regions due to the enhanced base stacking [38]. One of the approaches for monitoring of m^6^A presence at specific RNA positions was based on the melting properties of the RNA–DNA duplex in the presence of a modified nucleotide. The method developed by Golovina *et al*. [39] requires two oligodeoxyribonucleotide probes (carrying a quencher and a fluorophore) complementary to the analysed region (figure 3*a*). Analysis of fluorescence during melting of the produced duplex allowed detection of m^6^A at three specific positions of rRNA, one position of tRNA and one position of snRNA. The high specificity was also achieved in bulk cellular RNA. Although this method is simple to perform, it only has limited use due to the high concentration of the RNA sample required (0.4 µM). The possible use suggested by the authors could be screening of putative methyltransferase (MTase) knockout or knockdown cell lines in a search for unknown MTases [39].

Another approach for detecting of RNA methylation at specific positions used DNA hybridization probes that are sensitive to methylation of their complementary RNA sequences [40]. This method—termed ‘methylation-sensitive RNA fluorescence *in situ* hybridization’ (MR-FISH)—allows to monitor RNA methylation at specific sites in single cells. To achieve this, Ranasinghe *et al*. developed molecular beacons as hybridization probes sensitive to methylation in their complementary rRNA sequences in fixed cells. Another molecular beacon complementary to a remote non-methylated sequence in the same RNA strand was used for internal calibration (figure 3*b*). The method was shown to be sensitive to two adjacent m^6^_2_A bases, m^1^G and m^3^U. Interestingly, also the presence of m^6^A was shown to destabilize the duplex to some degree. Since this technique only requires simple equipment, it could be used in diagnostic tests for identification of antibiotic-resistant bacteria [40].

The base-pairing properties of m^6^A were used in another technique, in which the RNA template was reverse transcribed with deoxythymidine triphosphate analogue bearing a selenium atom at the 4-position (4SeT). The normal adenosine could base-pair with this analogue without any visible differences to A–T pairing, but the incorporation of 4SeT opposite m^6^A site was significantly stalled due to the perturbation of both hydrogen bonding and base stacking (figure 3*c*). The RT stalling took place for different reverse transcriptases used, but was affected by the reaction conditions, e.g. the incubation temperature or concentrations. To not only detect the presence of m^6^A at defined sites, but also to locate it for unknown samples, an m^6^A demethylase (FTO)-assisted strategy in combination with NGS was developed, resulting in m^6^A identification at single-nucleotide resolution. Up to date, the strategy was not applied for m^6^A identification in a real biological sample [41].

## 6. Approaches involving modification steps *in vivo* or in cells

Although most approaches for mapping RNA methylation sites in biological samples start from the RNA isolation from the cells/tissues, some approaches require a first step performed *in vivo* or in cells. Thus, one of the approaches for identification of direct targets of RNA cytosine MTases is based on the disruption of the methyl transfer by the suicide inhibitor 5-azacytidine (5-aza-C). For this, HeLa cells expressing an epitope-tagged m^5^C-RNA MTase DNMT2 were grown in presence of 5-aza-C, which was incorporated into nascent RNA [42]. Later the approach was used for identification and characterization of NSUN6 as a novel mammalian m^5^C tRNA MTase [43].

**Figure 4.**
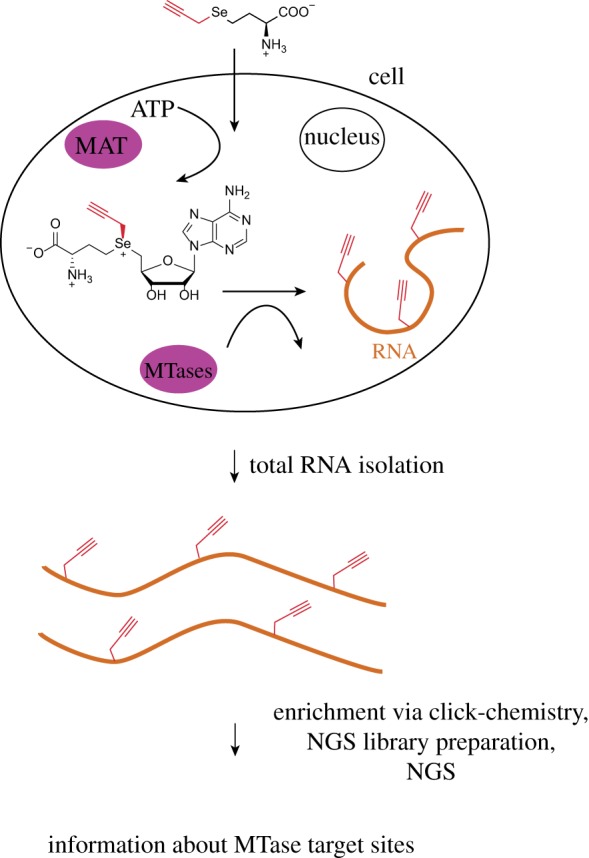
Metabolic labelling approach of MTase target sites. A synthetic methionine analogue bearing a propargyl group is taken up by HeLa cells, where it gets converted to a propargyl-bearing analogue of SAM by cellular methionine adenosine transferases (MATs). The cellular RNA MTases use the resulting SAM analogue to transfer the propargyl groups to their targets. After the isolation of total RNA, the modified RNA molecules can be enriched via click-chemistry, subjected to RT, NGS library preparation and NGS. Since the propargylation and subsequent clicking affects the RT signature at the modified position, the information about the MTase target sites can be achieved from the NGS data [45].

Most RNA MTases use *S*-adenosylmethionine (SAM) as a methyl donor, although some exceptions are known [44]. Recently, we established an approach for metabolic labelling of MTase target sites starting from methionine analogues [45]. Mammalian HeLa cells are fed with synthetic propargyl-l-selenohomocysteine, which gets converted by cellular methionine adenosyltransferases to a SAM analogue bearing a propargyl group instead of the methyl one (figure 4) [46]. Use of this cosubstrate by some cellular MTases results in the transfer of propargyl groups to the sites that are normally methylated. This is consistent with the *in vitro* results, showing that recombinant METTL3–METTL14 complex efficiently transfers the propargyl group to the *N*^6^-position in adenosine. Many other cellular MTases, including the ones from the eukaryotic 5′-capping machinery, also show a high level of promiscuity *in vitro* regarding the SAM analogues [47]. After the total RNA isolation, the transferred propargyl groups can be functionalized in a click-reaction with biotin azide, which enables enrichment of the RNA regions containing methylation sites on streptavidin beads. Based on additional *in vitro* data for RT in the presence of different *N*^6^-modifications and NGS, we anticipate that this method bears potential for detection of MTase target sites with single-nucleotide resolution [45].

Shu *et al*. [48] discussed the idea of MTase-assisted mapping of m^6^A sites with the help of SAM analogues. They showed that the METTL3–METTL14 complex (one of the known eukaryotic m^6^A MTases) transfers an allyl group from the corresponding SAM analogue, albeit with low yield. In this case, the RT signature is induced not via the click-chemistry, but via iodination of the allyl group, which leads to formation of *N*^1^,*N*^6^-cyclized adenosine. Labelling of the isolated total RNA in presence of METTL3–METTL14 and allyl-SAM could potentially be used in future for transcriptome-wide discrimination of A from m^6^A. For this, a much higher allyl transfer activity should be achieved [48].

## 7. Are universal NGS approaches for RNA methylation detection possible?

Many specialized NGS-based protocols were developed for detection of different RNA modifications. They often rely on the antibody recognition or on specific chemical treatment of the analysed modified base [8]. Owing to the great variety of naturally occurring RNA modifications, having a universal method for simultaneous site-specific detection of multiple modification would greatly benefit the field.

The NGS protocols greatly depend on the fidelity of the reverse transcriptase, which has long been known to be affected by the presence of different modifications. Recent studies aimed to systematically investigate the effect of several RNA nucleobase modifications on the performance of some commercially available RT enzymes. Thus, Potapov *et al*. [49] developed a method to study the effect of RNA modifications on the RT enzymes, which provides information on the frequency, type and sequence context of the RT errors. The effect of such modifications as m^6^A, m^5^C and m^5^U on the fidelity of M-MuLV, AMV and ProtoScript II enzymes was studied [49].

Another recent study hypothesized that different RNA modifications would cause distinguishable effects in deep sequencing data (patterns of mutations, truncations, insertions and deletions) and therefore elucidated the fingerprints of the modified RNA nucleobases [50]. Synthetic short RNA oligonucleotides carrying 10 base modifications were reverse transcribed with SuperScript IV and subjected to NGS sequencing. Under the used RT conditions, some modifications were statistically indistinguishable from the unmodified bases (m^5^C, m^5^U and m^6^A). Others (*N*^6^,*N*^6^-dimethyladenosine, m^1^A, inosine, m^1^G and *O*^6^-methylguanosine) yielded sequencing profiles distinct from adenosine and guanosine, respectively. However, it has to be noted that the polymerase responses varied depending on the sequence contexts. In addition, it is possible that modification-dependent patterns observed in this study may overlap with those of untested RNA modifications. Nevertheless, this study is an important proof of concept for simultaneous detection of several RNA modifications. In future, further investigations of the modification-specific RT signatures or protein engineering might lead to the discovery of an RT enzyme with more pronounced discrimination.

## 8. Use of third-generation sequencing for detecting RNA modifications

Third-generation sequencing, also known as single-molecule sequencing, is a class of diverse high-throughput sequencing technologies currently under development, whose working principles differ from NGS (often referred to as second-generation sequencing). In contrast to the latter, these emerging methods do not require breaking of the nucleotide strand to smaller segments nor amplification by PCR. Early experimental studies show the potential of third-generation technologies for detection of modified RNA nucleotides.

Nanopore sequencing detects single DNA or RNA molecules that are captured in the nanopore and translocated through it. To facilitate the capture and the sequencing, the RNA is ligated to a modular oligonucleotide adapter bearing a proprietary motor protein which regulates the RNA migration through the pore. A constant electric field is applied to the system, and the observed electric current is characteristically disrupted when a nucleotide passes through the pore, which makes the sequencing possible. The first attempt to read modified RNA nucleosides using direct nanopore sequencing was carried out in 2017 using the *E. coli* 16S rRNA [51]. One of the systematic base-call errors in the sequencing data corresponded to the known 7-methylguanosine position at G527. To prove that the error was caused by the presence of methylation, the reads from the wild-type strain were compared with the ones from the knockout strain lacking the enzyme responsible for the methylation at G527. As expected, the base-call error was eliminated in the knockout strain sample. This proved that the guanosine methylation alters the ionic current, thus making the nanopore detection of RNA methylation possible. Recently, another work showed the possibility of direct detection of RNA methylation with nanopore sequencing [52]. The perturbation of the current within the nanopore caused by m^6^A and m^5^C was studied for the synthetic RNA strands. The average current level was found to be locally perturbed near the modified positions. This preliminary observation was only made for two base modifications present in synthetic and fully modified RNA and is therefore not yet suitable for detection of RNA methylation in real biological samples. A higher sensitivity might be achieved in the future after further technological advances such as additional pore engineering.

Another emerging NGS technology is single-molecule real-time detection of reverse transcription (SMRT). Originally developed for DNA, it was also adapted for RNA sequencing. The RT of the RNA molecule is visualized due to the fluorophores attached to the terminal phosphate groups on the dNTPs. The removal of the label during the nucleotide incorporation allows for real-time monitoring of cDNA synthesis. The presence of m^6^A affected binding of the phospholinked nucleotide both in the synthetic RNA template and at the known site in mRNA when the HIV RT enzyme was used [53].

In summary, the field of detection of methylation sites in RNA is rapidly growing. New methods are in development not only for transcriptome-wide detection of unknown modification sites, but also for fast and easy monitoring of the presence of the methylated nucleoside at a given position. Most of the recent methods focus on the detection of m^6^A, probably due to its outstanding biological role. The future of the field is likely to be linked to the development of second- and third-generation sequencing technologies, which are now becoming more accessible.

## Data accessibility

This article has no additional data.
